# HDAC inhibitor misprocesses *bantam* oncomiRNA, but stimulates *hid* induced apoptotic pathway

**DOI:** 10.1038/srep14747

**Published:** 2015-10-07

**Authors:** Utpal Bhadra, Tanmoy Mondal, Indira Bag, Debasmita Mukhopadhyay, Paromita Das, Bibhuti B. Parida, Prathama S. Mainkar, Chada Raji Reddy, Manika Pal Bhadra

**Affiliations:** 1 Functional Genomics and Gene silencing group, CSIR-Centre for Cellular & Molecular Biology, Uppal Road, Hyderabad 500007, INDIA; 2 Centre for Chemical Biology, CSIR-Indian Institute of Chemical Technology, Uppal Road, Hyderabad 500 007, INDIA; 3 Division of Natural Products Chemistry, CSIR-Indian Institute of Chemical Technology, Uppal Road, Hyderabad 500 007, INDIA

## Abstract

Apoptosis or programmed cell death is critical for embryogenesis and tissue homeostasis. Uncontrolled apoptosis leads to different human disorders including immunodeficiency, autoimmune disorder and cancer. Several small molecules that control apoptosis have been identified. Here, we have shown the functional role of triazole derivative (DCPTN-PT) that acts as a potent HDAC inhibitor and mis-express proto onco microRNA (miRNA) *bantam.* To further understanding the mechanism of action of the molecule in apoptotic pathway, a series of experiments were also performed in *Drosophila*, a well known model organism in which the nature of human apoptosis is very analogous. DCPTN-PT mis processes *bantam* microRNA and alters its down regulatory target *hid* function and cleavage of Caspase-3 which in turn influence components of the mitochondrial apoptotic pathway in *Drosophila*. However regulatory microRNAs in other pro-apoptotic genes are not altered. Simultaneously, treatment of same molecule also affects the mitochondrial regulatory pathway in human tumour cell lines suggesting its conservative nature between fly and human. It is reasonable to propose that triazole derivative (DCPTN-PT) controls *bantam* oncomiRNA and increases *hid* induced apoptosis and is also able to influence mitochondrial apoptotic pathway.

In animal and plant kingdom, developing organs must be correctly patterned during which cell proliferation and apoptosis are coordinated through cell-to-cell communication. Cell proliferation, cell death and pattern formation are three linked important phenomena that occur sequentially during development. Apoptosis contributes to pattern development mainly by removing excess cell population and fine tuning developing structures. In normal cells, apoptosis gets initiated in response to developmental needs, whereas in cancer cells oncogenic signals lead to rapid and uncontrolled cell proliferation resulting in abnormal tissue growth^1^,^2^,^3^,^4^. How these cellular processes are coordinated during development is poorly understood and still remains a challenging task. In model organism *Drosophila*, a recently discovered tumour suppressor *hippo*-signalling pathway controls tissue growth by regulating cell proliferation, apoptosis and cell differentiation^5^,^6^,^7^,^8^,^9^,^10^,^11^,^12^,^13^,^14^. The *hippo* pathway acts as a key regulator for controlling organ size. It is often misregulated in various types of cancers. Several components of the *hippo* signalling pathways have been identified that are highly conserved in human^5^,^6^,^7^,^8^,^9^,^10^,^11^,^12^,^13^,^14^. In *Drosophila*, loss of *hippo* signalling results in overgrowth of tissues as the cells continue to proliferate and also show resistance to pro-apoptotic signals, which eliminates extra cells. So *hippo* pathway restricts cell proliferation and promotes apoptosis thereby controlling organ growth and tissue size. The *bantam* gene in *Drosophila* produces a 21 nt long microRNA (miRNA). Its expression is temporally and spatially controlled in response to patterning cues. It acts as a downstream target of *hippo* signalling pathway that affects tissue size^5^,^6^,^7^,^8^,^9^,^10^,^11^,^12^,^13^,^14^,^15^. Tissues with over expressed *bantam* miRNA are always larger than normal tissues. The mutated *bantam* oncomiRNA show an opposite effect. The *bantam* oncomiRNA prevents apoptosis by controlling downstream pro-apoptotic target *head involution defect (hid)* and cleavage of Caspase-3 in programmed cell death.

MicroRNAs (miRNAs) are tiny non-coding endogenous RNAs that are involved in gene regulation of many developmental processes. Thousands of miRNAs have been identified in human and other organisms that control nearly 30–40% of the total genes^16^,^17^. Processing of miRNA is tightly maintained both temporally and spatially^18^,^19^,^20^,^21^. During biogenesis, nearly 70 nt long precursor miRNA that forms imperfect hairpin like loop is cleaved by a RNase-III enzyme *Dicer1* to form short mature microRNA^22^,^23^. Typically, the antisense arm of the hairpin structure (pre-miRNA) recovers as mature miRNA^24^,^25^,^26^,^27^. Mis-regulation of biogenesis is associated with various diseases including auto-immune disorders and cancer.

Small molecules that target miRNAs are a novel approach to find better therapeutics for cancer. Recently, several *in vivo* screening platforms have been developed to search small molecules that mis-regulate the processing of microRNA. Though a group of inhibitors have been identified that interfere with microRNA processing^28^,^29^, but a platform for chemical analogues that alter oncomi RNA processing in cancer is still missing. Cellular inhibitor that misprocesses oncomiRNA for inducing apoptosis or programmed cell death pathway including endogenous pattern formation has tremendous potentiality as therapeutic agent in cancer. Therefore, development of transgenic model for performing *in vivo* screening of small molecules provides the platform for understanding the potential insight of apoptotic pathway and their molecular function at every step allowing the only optimal choice for evaluating the functionality of each molecule.

Several inhibitors of apoptosis have been identified and characterized in recent days. Here we have made an attempt to find an inhibitor that specifically targets microRNAs that affect pro-apoptotic genes. Out of our six synthesized HDAC inhibitors (Fig. 1) only compound-2 showed a distinct modulation on *bantam* microRNA processing but not in other microRNAs that regulate major pro-apoptotic genes *reaper*, *grim* and *Drice* etc. Here we demonstrate the role of *bantam* microRNA and its pro-apoptotic target *hid* to understand in-depth action mechanism of compound-2 in controlling apoptosis.

## Results

### Synthesis and biological evaluation

Numerous compounds having heterocyclic frameworks have been explored to find lead for developing better therapeutics. In continuation, a library of sixty-three 1, 2, 3-triazoles with substitutions at either 4—or 4, 5-positions were synthesized and screened for their biological activity. One of the initial screenings indicated these scaffolds as HDAC inhibitors. To explore the activity, a set of six triazole derivatives were synthesized from azide derived from either advanced, intermediate or the drug itself. We have selected sertraline intermediate and ibuprofen as the azide precursors, keeping the idea that their protein binding properties have been well studied.

The triazoles, **1–6**, were synthesized using Huisgen’s 3 + 2 cyclo-addition reaction or ‘click chemistry’. The Cu catalysed reaction lead to the formation of substituted-1, 2, 3-triazoles. Thus, azide precursor obtained from intermediate for sertraline was reacted with alkynes such as phenyl-acetylene, 1-heptyne and acetylene dicarboxylic acid methyl ester in presence of Cu catalyst to obtain the corresponding triazoles **1** to **3**, in good yield 88–98%. For the next series, Ibuprofen was converted to an azide derivative, which upon reaction with the similar acetylenes used above resulted in triazoles **4, 5** & **6** in 98, 95 and 94% yields, respectively. All the triazole derivatives **1–6** were fully characterized (For experimental details and spectral data, see Supporting Information).

**Figure 1:**
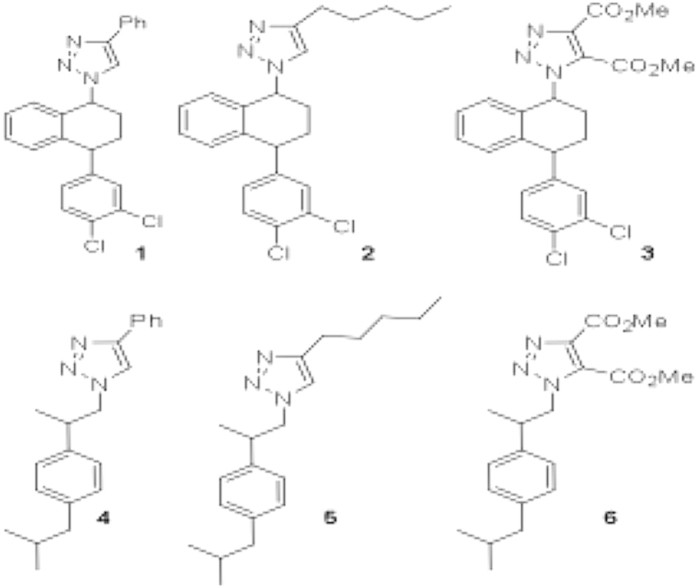
Chemical structures of triazole derivatives and DCP TN-PT compound.

The triazole derivatives (**1–6,** Fig. 1) obtained from these azides proved encouraging as they exhibited cell cycle arrest at G1 phase with significant cell death (4 μM conc.) when administered to *Drosophila* S2 or proliferating tumor HeLa cells (Fig S1-S2).

These compounds (**1–6**) were further subjected to five dose screening (1 μM, 2 μM. 3 μM, 4 μM and 8 μM) assay followed by cytotoxicity assays. The 1-(4-(3, 4-Dichlorophenyl)-1, 2, 3, 4-tetrahydronaphthalen-1-yl)-4-pentyl-1*H*-1, 2, 3-triazole (Compound **2,** DCPTN-PT)was found the most potent with respect to cell cycle arrest (G1 phase) and induction of apoptosis(50% cell death, 4 μM and 8 μM conc). A proportional increase in the accumulation of cells at sub G1 phase was observed with the increase in the concentration of the compound (Fig S3-S4). Cytotoxicity MTT (MTT = 3-(4, 5-dimethylthiazol-2-yl)-2, 5-diphenyltetrazolium-bromide) assay showed that DCPTN-PT produced nearly 50% death to HeLa cells (IC_50_ value = HeLa 3.4469 μM) and S2 cells (*Drosophila melanogaster*) when administered at a concentration of 4 μM (Fig S1-S2).

### HDAC assay

**Figure 2:**
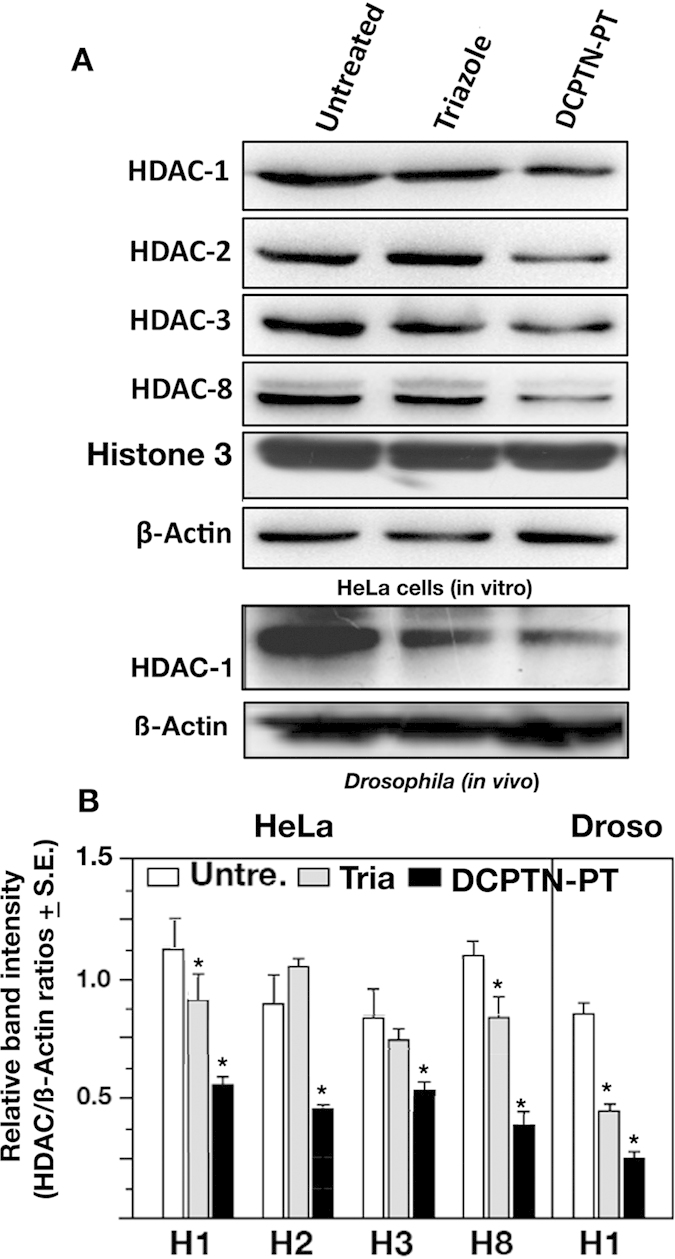
Quantitative Western analysis for estimation of HDAC proteins in HeLa cells and live *Drosophila* larvae. (**A**) Proteins were extracted from untreated cells and from cells treated with Triazole derivative and DCPTN-PT (4 μM/ml conc). Western analysis was performed by hybridizing with different HDAC proteins. The histone 3 and ß-Actin were used as loading control. Same western blots analysis was performed with live *Drosophila* mature larvae (*in vivo*). Proteins were extracted from unfed and DCPTN-PT fed larvae. Blots were probed with HDAC 1 and counter probed with ß-Actin as a gel loading control. (**B**) Relative band density from each lane was calculated from three separate gels. Mean ratios and S.E. was calculated and plotted as the Bar diagram. The values with asterisks are significantly different from that of the untreated /unfed control at the 95% confidence level (P < 0.05).

HDACs have shown to be promising targets for cancer as they regulate transcription and triazole scaffolds have shown to play some role as HDAC inhibitors^30^. To test whether the triazole scaffold in the compound produce any inhibitory effect on HDAC proteins, series of quantitative western blots were performed. Human cervical cancer cells (HeLa) and *Drosophila* S2 cells were treated with different concentration of DCPTN-PT compound (1–4 μM) for 72 hours followed by HDAC estimation. Untreated cells served as control. The mean ratios of HDAC proteins relative to ß-Actin protein were measured in three independent assays and plotted in bar histogram. It was found that all four HDACs (HDAC 1, 2, 3 and 8) were reduced consistently upon treatment of HeLa cells with DCPTN-PT. Therefore, DCPTN-PT treatment produced a significant reduction of HDAC 1, 2, 3 and 8 proteins in HeLa cells (Fig. 2A).

To see whether a similar effect on HDAC inhibition occurs in live model organism also, *Drosophila* larvae were allowed to feed on culture media containing the compound DCPTN-PT (12 μM in 3 ml food media). Western blots were performed with proteins isolated from unfed and compound fed larvae to examine the effect on HDACs. A similar level of reduction of HDAC1 was found in live *Drosophila* larvae after feeding on the compound in the culture medium (12 μM in 3 ml food media) (Fig. 2A). Therefore, DCPTN-PT acts as a potential HDAC inhibitor both in human tumour cells (HeLa) and *Drosophila* larvae *in vivo* (Fig. 2B). Overall these findings through *in vitro* and *in vivo* assays suggest that DCPTN-PT acts as a potent HDAC inhibitor.

HDAC inhibitors are known to enhance apoptosis, growth arrest and differentiation^31^. They have positive effect on tumour suppressor pathway^32^,^33^. In this study, we evaluated the role of this newly synthesized HDAC inhibitor (DCPTN-PT) that induces apoptosis pathway in the *in vivo* model *Drosophila*.

*Drosophila* model organism has several advantages. Its rapid life cycle allow extensive studies with transgenics for several generations. Moreover, human oncomiRNA are extremely analogous to *Drosophila*^34^.

### Apoptosis symptoms and screening for pro apoptotic gene regulation

**Figure 3:**
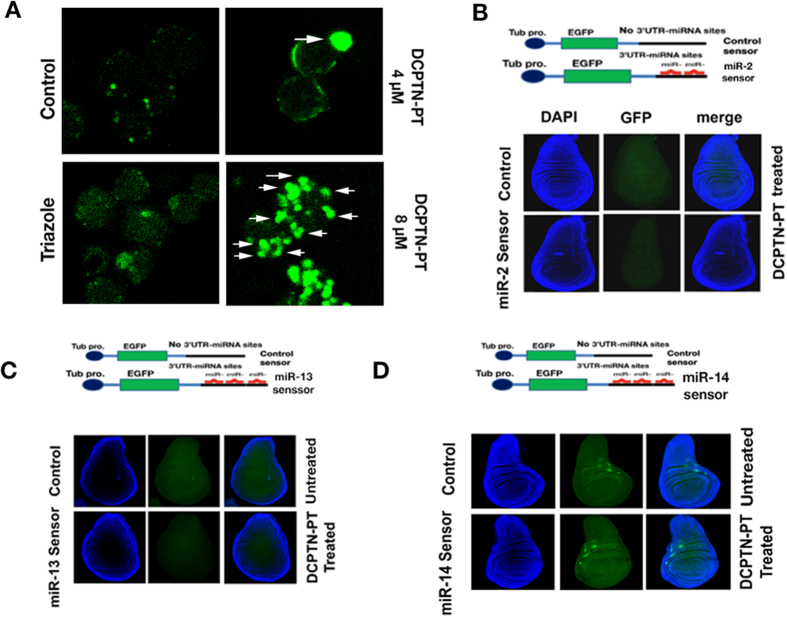
(**A**) Effect of DCPTN-PT conjugates results in DNA fragmentation of S2 cells. The white arrow indicates the site of DNA fragmentation. (**B**–**D**) Schematic diagrams of control and different micro RNA sensors. Larval wing discs expressing the tubulin-EGFP reporter gene imaged at identical confocal microscope setting. The genotype of each larva is noted.

To investigate generalized apoptosis features, the DCPTN-PT treated S2 cells were analyzed by conventional TUNEL assay. The assay analyzed different apoptosis signatures including chromatin condensation, DNA fragmentation, cellular shrinkage and blebbing etc. These results showed that DNA fragmentation was more prominent when S2 cells were administered with 8 μM of DCPTN-PT compound compared to untreated controls (Fig. 3A). The DNA fragmentation as seen by TUNEL assay was concentration dependent. At 4 μM concentration of DCPTN-PT, the fragmentation was lesser than what was observed at 8 μM of compound in each nucleus of *Drosophila* S2 cell. However the precursor molecules traizole did not induce DNA fragmentation at similar intensity.

Micro RNAs play a role in cancer progression by regulating different pathways of genes that affect apoptosis such as *p53, myc* etc. Since DCPTN-PT is a potent molecule in the process of apoptotic pathways, the regulatory microRNA (miR) elements of major pro-apoptotic proteins were tested. A novel transgenic *Drosophila* approach was employed. The transgenic construct (micro RNA line) expresses Enhanced Green Fluorescent Protein (EGFP) under the control of *tubulin* promoter. The construct is fused with two to three complete micro RNA target sequences at the 3′UTR. A comparable construct without miRNA target sequences at the same gene end served as control (control sensor). Three transgenic constructs each carrying microRNA coupling sequences that are linked to endogenous regulatory domains at the 3′UTR of three different pro-apoptotic genes *reaper*, *grim* and *drice* were screened. Rearing of larvae from each of three different transgenic stocks in DCPTN-PT containing media did not produce any enhancement of the expression of reporter EGFP relative to control sensor (Fig. 3B–D). Therefore, feeding of the DCPTN-PT containing culture media did not depress the regulatory microRNAs (miR-2, miR-13 and miR-14) that control the translation of three pro-apoptotic host genes (miR-2 and miR-13 targets *rpr*, *grim* and miR-14 targets *Drice*), suggesting that DCPTN-PT compound is not an inhibitor of all proapoptotic genes but very specific to oncomiR *bantam*.

### The bantam miRNA assay in transgenic fly

**Figure 4:**
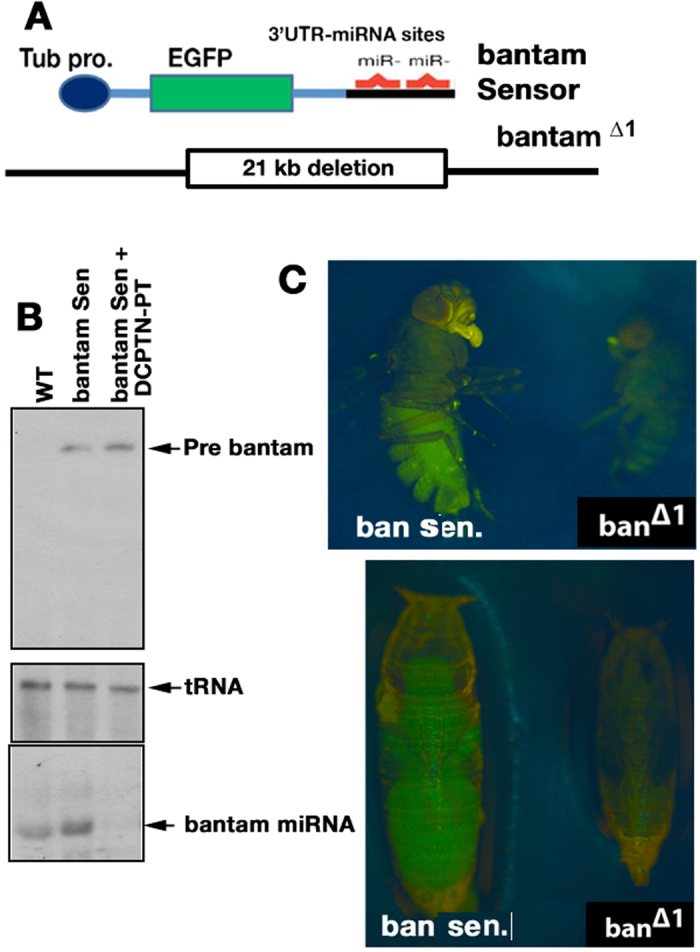
(**A**) Diagram of bantam sensor and functional bantam deletion null allele (bantam Δ1) are noted (**B**) Autoradiograms showing Northern blot for *bantam* pre-miRNA and mature miRNA in different larvae. The genotypes of larvae were noted. tRNA, used as loading control. (**C**) Continuous feeding to EGFP bantam sensor (ban sen.) and bantam Δ1 deletion mutant (*ban^Δ1^*) larvae were grown to pupae and adults. Sensor organisms after feeding expressed EGFP under epifluorescence images.

*Drosophila bantam* gene encodes a 21 nucleotide miRNA that promotes cell proliferation and reduces apoptosis. It is interrelated with tumour suppressor *hippo* pathway and *hid* induced apoptosis pathway in cancer. Endogenous removal of the *bantam* gene enhances *hid* induced apoptosis^15^. Therefore *hid* pro apoptotic gene acts as a target for regulation by 21 nucleotide *bantam* miRNA. This study not only allows the understanding of mechanism of action of the molecule in the apoptotic pathway, but also provides a unique platform for *in vivo* screening of molecules that induces apoptosis for developing cancer therapeutics. Based on the modulation of apoptotic signature and non significant effect on major pro-apoptotic genes, we tested, whether DCPTN-PT mis-expresses *bantam* oncomiRNA or not. We fed separate batches of larvae with food containing DCPTN-PT (12 μM in 3 ml food).These larvae carried either a control or *bantam* miRNA transgenic construct. Wild type *Drosophila* larvae were also fed with the same concentration of DCPTN-PT that serves as control. Moreover, a construct carrying a large deletion of the chromosomal fragment of the *bantam* endogenous locus was used as null allele (*bantam^Δ1^*)^15^ (Fig. 4A). Therefore in the sensor line, *bantam* miRNA binds to the target sequence at the 3′ end of the trans gene and blocks EGFP expression. Similar to earlier report^15^ the expression was clearly found in the larval wing discs, a tissue mass that develops into adult wings and proliferating brain cells. It is noted that *bantam* miRNA is expressed at all developmental stages in varying levels. To confirm the sensitivity of the transgenic stocks we performed Northern blot analysis by isolating total RNA from larvae of different lines. In wild type larvae, a *bantam* RNA of 21 nt long was expressed normally whereas in *bantam* sensor transgenic line, pre microRNA (70 nt) and processed mature microRNA (21 nt) were enriched. Interestingly feeding of DCPTN-PT compound to the same *bantam* sensor larvae did not produce even a trace amount of mature *bantam* RNA. The transfer RNA (tRNA) in each lane of the Northern hybridization was used as a gel loading control (Fig. 4B). This confirmed that continuous feeding of DCPTN-PT to *Drosophila* larvae misprocesses the synthesis of *bantam* oncomiRNA.

We also performed a series of rescue assays by feeding DCPTN-PT to *bantam* sensor and *bantam*^*∆1*^ transgenic stocks. The deleted (*bantam*^*∆1*^) homozygous pupae lack some or all imaginal discs and show undergrowth. Continuous feeding of DCPTN-PT to *bantam* sensor showed normal growth of homozygous adult and pupae relative to unfed *bantam*^*∆1*^ mutants (Fig. 4C). The *bantam* sensor expresses EGFP protein in adult flies.

**Figure 5:**
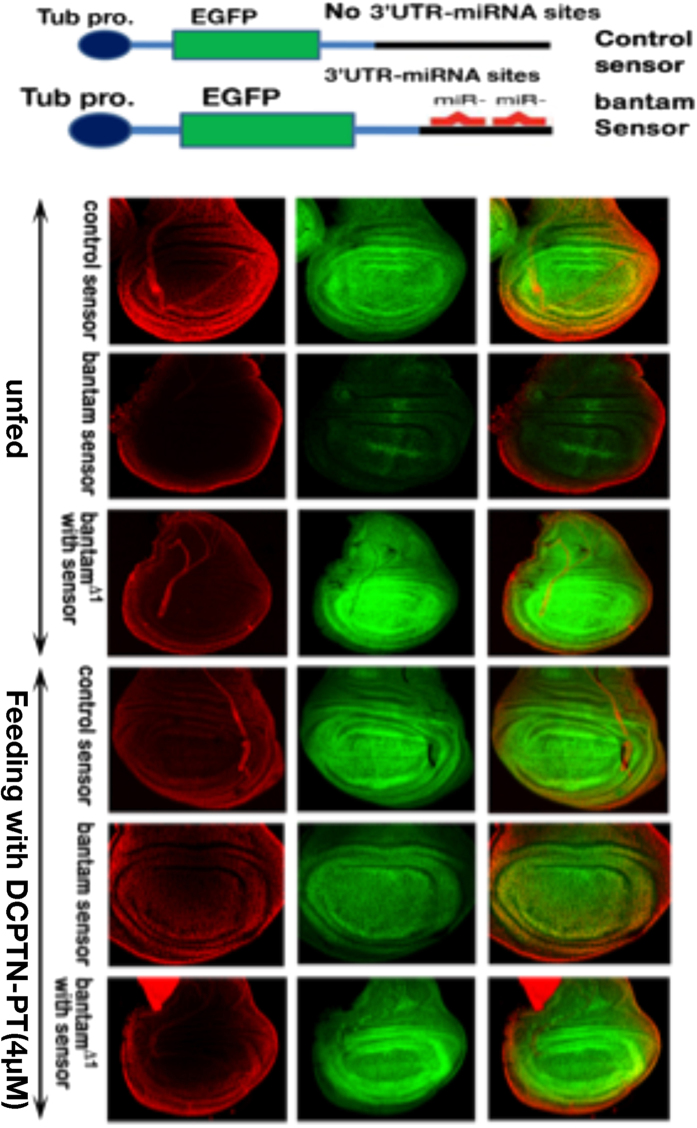
Schematic diagrams of control and bantam sensor. Larval wing discs expressing the tubulin-EGFP reporter gene imaged at identical confocal microscope setting. The genotype of each larva is noted.

The HDAC inhibitor, DCPTN-PT was further tested against the *bantam* miRNA, by feeding to control, sensor and mutant transgenic larvae. The level of EGFP expression in larval wing discs was observed. Low EGFP expression compared to control was observed in DCPTN-PT unfed sensor and larval discs (Fig. 5). Interestingly, EGFP expression was strongly increased upon feeding in bantam sensor lines. No change in EGFP expression was seen upon feeding of the same compound neither in control sensor nor in the mutant *bantam*^*∆1*^ lines (Fig. 5). Therefore DCPTN-PT not only acts as a HDAC inhibitor but also reduces *bantam* oncomiRNA activity simultaneously to increase EGFP expression in the larva of sensor line. In other words, DCPTN-PT feeding blocks *bantam* oncomiRNA production in the wing discs, enhancing the EGFP expression.

### Hid expression and apoptosis

Previous studies have shown that *bantam* miRNA has three independent targets in 3′UTR of the apoptosis-inducing gene *head involution defect (hid)*^15^. The conservation of these sequences suggests a functional relationship between *bantam* miRNA and target gene *hid*. Continuous feeding of DCPTN-PT compound misprocesses the normal *bantam* mature microRNA biogenesis. Defective biogenesis reduces *bantam* microRNA production. Therefore, less *bantam* miRNA production may not be sufficient to bind to 3′ end of the target gene *hid*, thereby leading to translational blockage for the production of Hid protein significantly.

**Figure 6:**
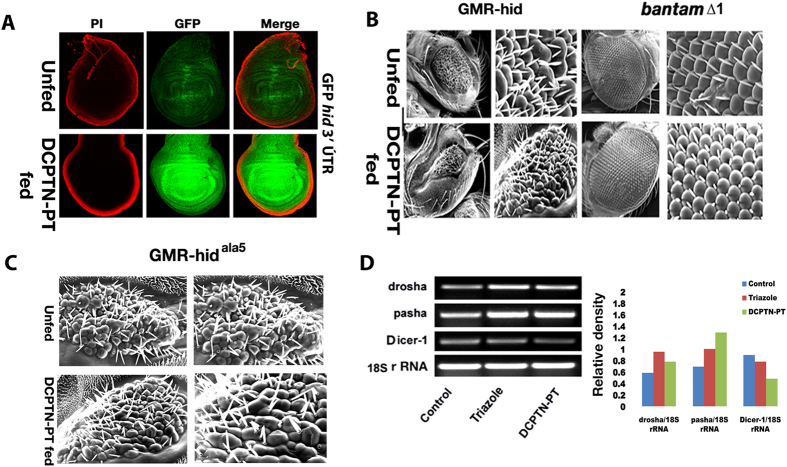
(**A**) Different wing disc showing expression of the tubutin-EGFP transgene with the hid 3′UTR **(B**) Scanning electron microscopy (SEM) of adult fly eyes and ommatidia structure in GMR-*hid* fusion transgene construct and bantam^Δ1^ mutant as noted. Enlarged view of the adult eyes is displayed. Adult flies of *GMR-hid* lines emerging from DCPTN-PT fed larvae develop smaller rough eyes when compared to unfed controls indicating apoptosis. Flies emerging from deleted bantam^Δ1^ stock do not show any significant effect (**C**) SEM figures of adult eyes of *GMR-hid (Ala5)* hybrid construct. No significant change in size or morphology is observed in flies emerging from compound fed larvae as compared to unfed controls. (**D**) The gel figures were shown for quantitative measurement of different transcript of core microprocessor genes relative to loading control. Three different S2 cells were used. S2 cells were incubated with precursor triazole and DCPTN-PT compounds separately. The relative density of each band was calculated relative to loading control band and the mean ratios were presented by a bar diagram.

To assess whether there is an effect on the *hid* gene, we fed the compound to transgenic larvae that contained predicted *bantam* target sites (*tubulin*-EGFP reporter transgene fused to the 3′UTR on the *hid* mRNA). The resulting EGFP pattern observed in the wing disc was identical to that produced by the *bantam* sensor. Similar to *bantam* sensor, *tubulin*-EGFP-*hid*3′UTR sensor showed an immense up regulation of EGFP expression, when larvae were fed with DCPTN-PT (12 μM in 3 ml of food) containing culture media (Fig. 6A). It demonstrated that feeding of DCPTN-PT blocked the production of mature *bantam* oncomiRNA and leads to *hid* up regulation. Thus *bantam* miRNA can block expression of a transgene containing the *hid* 3′ UTR in *tubulin*-EGFP reporter.

To analyse the effect on adult stage DCPTN-PT containing culture media was fed to transgenic larvae that carry a Glass Multimer Report (GMR) promoter fused to *hid* and allowed them to grow into adult flies. GMR promoter is particularly expressed in eye and eye precursor tissues (imaginal disc). This unique stock carrying an eye specific promoter allows any modulation of the specific pathway to be visualised with the change of eye phenotype^15^. Induction of cell death in post mitotic cells of the eye imaginal disc in GMR *hid* transgene flies produced small rough eye phenotype in adults^15^. The eye size was further reduced after continuous feeding with DCPTN-PT containing culture media to the larvae that developed into adults indicating a dramatic increase of apoptosis. Moreover, ommatidital structure was largely irregular and drastically reduced. Interestingly no distortion was found in the eye phenotype and ommatidial structure of adult flies that emerged from *bantam*^*∆1*^ (stock in which the *bantam* microRNA sequences were totally deleted) larvae fed with DCPTN-PT containing culture media (Fig. 6B). To further reconfirm the specificity of the molecule on *hid* induced apoptosis in the adult flies of *GMR-hid* lines, we fed the same concentration of the compound to another transgenic line *GMR-hid (Ala5*) that contains 5 conserved Extracellular Signal Regulated Kinase (ERK) phosphorylation sites mutated to alanine^15^ and thereby unable to suppress the activation of the ERK-Mitogen-activated Protein Kinase (MAPK) pathway^35^. This transgenic line induces cell death in post mitotic cells in adult eye producing small rough eye phenotype. Continuous feeding of the compound to larvae of *GMR-hid (Ala5)* did not produce any change in the size of the eye phenotype nor ommatidial arrangements or structures (Fig. 6C). Though both the transgenes *GMR-hid* and *GMR-hid (Ala5)* lead small rough eye phenotype, suppression of the *GMR-hid* is stronger than *GMR-hid (Ala5)* (Fig. 6C). The ommatidial structure was largely reduced by continuous feeding of compound in *GMR-hid* but not in the *GMR-hid (Ala5).* It suggests a more specific increment of endogenous *hid* activity and DCPTN-PT molecules cannot activate endogenous *hid* by the common ERK-MAPK pathway.

To further address the issue whether misregulation of *bantam* by the DCPTN-PT up regulate endogenous *hid* expression in larvae, a quantitative Western blot analysis was performed with different genotypes. In control and *bantam* sensor larvae *hid* expression was relatively less when compared to the expression seen in larvae fed with DCPTN-PT at two different concentrations. Thus expression of endogenous *hid* was up regulated upon feeding the compound leading to over expression of the HID proteins. To eliminate the oversaturation of Western blot hybridization we also used low concentration of compound (2 μM) in the quantitative western blot analysis. A similar trend of over expression of the target protein was observed after feeding the compounds at two different concentrations indicating that oversaturation of the gel blots do not interfere the over expression.

Though there was a significant increment in the production of HID proteins by the reduction of *bantam* miRNA, as a result of continuous feeding of DCPTN-PT molecules, there was no change in the *hid* mRNA expression (Fig S5). This indicates that the over expression of the HID protein due to repression of *bantam* miRNA is probably due to reduction in the translation blockage of the *hid* mRNA.

In search of whether misregulation of *bantam* miRNA is a general defect of microRNA processing or specific for *bantam*, we have estimated the amount of core genes that are involved in microRNA processing. Earlier it was found that Dicer-1 in *Drosophila*, which is supposed to cleave the mature microRNAs, has no effect by its mutation to the modulation of all microRNAs. Based on such specificity, we can assume that DCPTN-PT might be affected only on *bantam* misexpression. To ensure that core microRNA processor genes were measured by quantitative RT PCR. It was found that DCPTN-PT compound and precursor trialzole compound has no significant effect on *drosha* and *pasha* RNA when S2 cells were incubated with two separate compounds (DCPTN-PT and precursor Triazole) (Fig. 6D). However their effect in *Dicer1* transcripts is marginal but significant. These findings showed that the effect of DCPTN-PT is very specific and can be potent candidate for *bantam* regulated process.

### Effect of DCPTN-PT in Mitochondrial pathway

**Figure 7:**
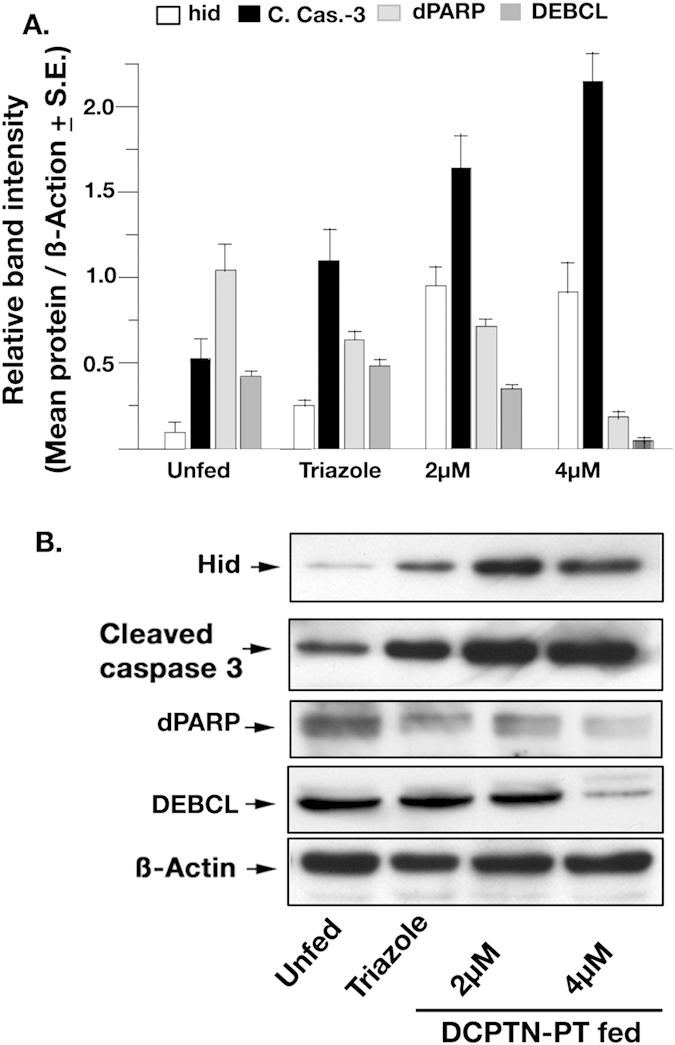
Bar diagrams representing Western blot analysis of different proteins. (**A**) Bars are drawn from the mean ratios as estimated by the band intensities of each protein relative to ß-Actin loading control. The mean ratios + S.E. from triplicate identical gels were calculated for each bar. (**B**) Panel representing series of western blot gels. The feeding of the *Drosophila* larvae is noted at the bottom. The proteins were noted at the left side of the panel. The ß-Actin lane acts as loading control.

We next estimated the protein levels of the known apoptotic marker, Caspase-3. Total proteins were extracted from both DCPTN-PT fed and unfed larvae and western blot analysis was performed with antibody against cleaved Caspase-3. The level of apoptotic proteins increased considerably upon feeding when compared to unfed controls (Fig. 7A,B).

Two main classes of apoptosis are the mitochondrial extrinsic and intrinsic pathways. Both involve caspase activation that lead to the cleavage of multiple intracellular substrates. To address that the activation of Caspase-3 also influences *Drosophila* intrinsic pathway, we estimated the protein level of *Drosophila* Bcl-2 family, especially pro-apoptotic protein DEBCL and *Drosophila* Poly ADP Ribose Polymerase (dPARP) another effector protein in the pathway, after continuous feeding of DCPTN-PT molecules to larvae. Continuous feeding of larvae with DCPTN-PT at two different concentrations significantly reduced the level of expression of both the proteins DEBCL and dPARP relative to the triazole fed and unfed larvae (Fig. 7A,B). The Bcl-2 family proteins and PARP are highly conserved throughout evolution^36^. To ensure that DCPTN-PT molecules produce similar effect in mammalian cell lines; we performed the same experiment after incubating HeLa cells with DCPTN-PT molecules. A similar trend was found (Fig. S6) indicating that incubation of DCPTN-PT in two different organisms does not causes any effect on the conserved homology among species.

## Discussion

We identified a novel function of a newly synthesized triazole derivative that reduces drastically a critical oncomiRNA from *bantam* coded gene responsible for promoting cell proliferation and blocking apoptosis^15^. Mode of action of DCPTN-PT molecule in depth was evaluated functionally. The misexpression of proto-oncomiRNA, *bantam* might be responsible for diseases of cell proliferation that inhibit programmed cell death in *Drosophila* and vertebrates. The DCPTN-PT dependent misregulation of *bantam* microRNA reduces tissue growth unlike *bantam*^*∆1*^.

To investigate how misprocessing of *bantam* microRNA would promote cell death and prevent rapid cell division, it is necessary to determine it’s down regulatory genes. It was shown that *bantam* microRNA control pro-apoptotic gene *hid* directly^15^. In this study, it is found that DCPTN-PT acts as a negative regulator for *bantam* microRNA that reduces mature miRNA dramatically and increases target protein level (as HID) that might be a positive regulator of cell apoptosis or cell death. The reduction of *bantam* microRNA by the continuous feeding of DCPTN-PT may not be sufficient at the 3′ UTR endogenous binding of the *hid* locus. As a result, Hid proteins increase in amount by breaking *bantam* miRNA translation blockage of *hid* 3′ UTR. We also found that DCPTN-PT affects mitochondrial apoptotic pathways of *Drosophila* and mammalian cell lines. It suggests that DCPTN-PT orchestrates multifactor aspects of apoptosis, apart from degradation of *bantam* microRNA prominently, but also affects mitochondrial apoptotic pathway. It therefore seems likely that DCPTN-PT may reduce *bantam* oncomiRNA through *hid* mRNA and a yet to be identified positive regulator of cell death via Caspase-3 dependent pathway. In depth characterization of DCPTN-PT will show additional predicted targets that determine how *bantam* regulates apoptosis.

It is noteworthy that triazole analogues stimulate programmed cell death and pattern formation. This also provides a unique *in vivo* platform for screening molecules that interfere with micro RNA pathway.

**Figure 8:**
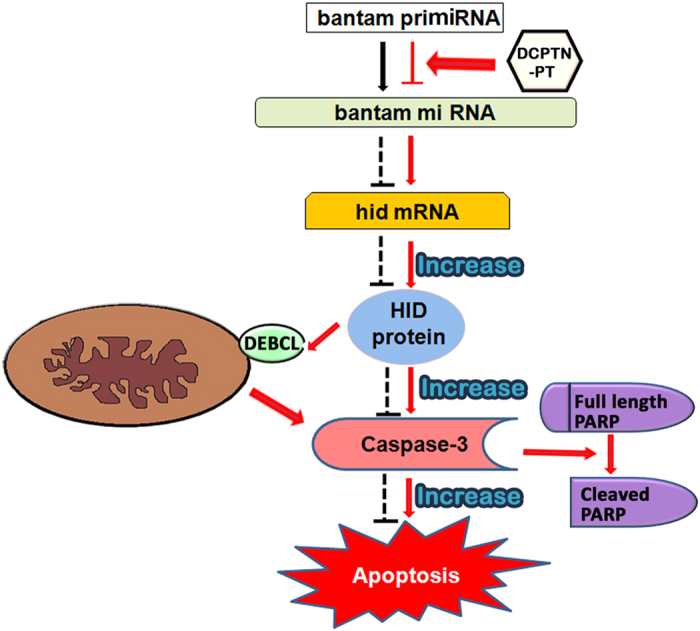
Schematic model of the pathway of action of DCPTN-PT in apoptosis. Red arrow represents feeding of DCPTN-PT increase apoptosis.

In conclusion an in-depth chemical *in vivo* screening method was been developed that not only identifies small molecules as a defective microprocessor of oncomiRNA, but also classifies their elaborate biological functions in cell and development pathways. Here changes in *bantam* activity introduced by triazole analogues developmentally up regulated programmed cell death in model organism (Fig. 8). Therefore, small molecules can function as unique modulator for understanding the role in misprocessing of cell proliferating *miRNA* and enhance apoptosis that probably open a new door for alternate use of natural chemical for regulating oncomiRNA.

In higher animals many programmed cell deaths are characterized by cell apoptosis. However DCPTN-PT fills a significant gap in our understanding of the caspase dependent apoptosis. In particular we have less information concerning how Caspases initiate membrane changes that are required for phagocytosis of the dying cells, further experiments would help us to unravel the understanding of how cells coordinate and shape the response to the disposal of dying cells. However DCPTN-PT molecules restrict their function to determine the Caspase participation in other cellular events such as differentiation and pattern formation. It also proposed that DCPTN-PT provide a positive link between the apoptotic mechanism that regulates patterning and tissue growth during animal development.

## Methods

### Northern Blots

Total RNA was resolved on 15% denaturing acrylamide gels and hybridized with 5′ end labelled oligonucleotide probes as follows. A tRNA probe was used as a loading control as described ^[1]^ *Drosophila bantam 5*′ end labelling probe ACAAGTGAGATCATTTTGAAAGCTGATTTTGT.

### Western Blots

For Western blot, freshly dissected wing imaginal discs were homogenized in lysis buffer [6% SDS, 1 mM EDTA, 2 mM PMSF, 10 μg/ml Aprotinin, 10 μg/ml Leupeptin, 10 μg/ml Pepstatin] and boiled at 95 °C for 5 minutes. Total protein was isolated and estimated using Bradford assay. Quantitative Western blot analysis was carried out using anti-rabbit EGFP (1:1000), Hid (1:500), cleaved caspase 3 (1:1000) antibodies. The blots were re-probed with anti-mouse ß-Actin (1:2000) as a gel loading control. For Histone estimation Histone 1, 2, 4, 8 (1:1000) antibodies were used. For loading control, Histone 3 (1:2000) dilution was used. For quantitative Western blot analysis from untreated and treated cell lines, total proteins were extracted from 5–10 × 10^5^ HeLa cells. Hybridization was conducted with mammalian Cleaved PARP (1:1000 dilution) Full length PARP (1:1250), Active caspase-3 (1:1500) Caspase 9 (1:1000). Cytochrome c (1:1500), Bax (1:1000) and Bcl-2 (1:1000) antibodies. After washing, the blots were incubated with secondary antibodies conjugated to horseradish-peroxidase. Immunoreactive bands were detected by enhanced chemiluminescence (ECL). Membranes were stripped and incubated with anti-mouse ß-Actin (1:2000) antibody to ensure equal protein loading.

### Real time PCR analysis

Total RNA was isolated by TRIzol® (Invitrogen, USA) method. RNA concentration & purity were measured by Nanodrop. RNA clean up and DNase treatment were performed to get DNA free pure RNA. cDNA was synthesized using DNase-treated pure total RNA. Real-time RT-PCR analyses were carried out using Applied Biosystem Power SYBR® Green PCR Master Mix in 7900HT Fast Real-Time PCR System (Applied Biosystem, USA). Reaction mixture was prepared using following components (reaction volume –20 μl)—6 μl PCR-H2O, 1 μl forward primer (0.5 μM), 1 μl reverse primer (0.5 μM), 10 μl Power SYBR® Green PCR Master Mix and as a template 2 μl cDNA (50 ng) was added. Using the following protocol amplification and quantification procedure were carried out- Initial denaturation (95 °C for 1 min), amplification and quantification program repeated 40 cycles (95 °C for 10 s, 58 °C for 10 s, 72 °C for 20 s with each step fluorescence measurement mode), melting curve program (55 °C–95 °C) and finally cooling to 40 °C.

The following primers were used for amplification

hid primers

RT-Forward primer—5′ TTCCTGCCCTCTTTCTTTG 3′

RT-Reverse Primer—5′ GTCCTTATCCGCTTCCTTCC 3′

18s RNA primers

Forward: 5′- CCTTATGGGACGTGTGCTTT -3′

Reverse: 5′- CCTGCTGCCTTCCTTAGATG -3′

## Additional Information

**How to cite this article**: Bhadra, U. *et al.* HDAC inhibitor misprocesses *bantam* oncomiRNA, but stimulates *hid* induced apoptotic pathway. *Sci. Rep.* **5**, 14747; doi: 10.1038/srep14747 (2015).

## Supplementary Material

Supplementary Information
