# Supplementary material for: HDAC inhibitor misprocesses bantam oncomiRNA, but stimulates hid induced apoptotic pathway
Source: Sci Rep. 2015 Oct 7;5:14747. doi: 10.1038/srep14747 (PMC4595805; doi:10.1038/srep14747)
Supplement: Supplementary Information [file srep14747-s1.doc]

**Supplementary online Information**

HDAC inhibitor misprocesses *bantam* oncomiRNA, but stimulates *hid*  induced apoptosis pathway.

Utpal Bhadra^1^*, Tanmoy Mondal^2^, Indira Bag^1^, Debasmita Mukhopadhyay^2^, Paromita Das^2^, Bibhuti B. Parida^3^, Prathama S. Mainkar^3^, Chada Raji Reddy^3^, Manika Pal Bhadra*^2^

*^1^Functional Genomics and Gene silencing Group, Centre for Cellular and Molecular Biology (CSIR)Hyderabad, Índia 500007. Tel: 91-40-27192513: Fax: 91-40-27160531*

*^2^Centre for Chemical Biology*, *Indian Institute of Chemical Technology (CSIR), Hyderabad, India. Tel: 91-40-27193210; Fax: 91-40-27160512*

*^3^Organic Chemistry Division-I, Indian Institute of Chemical Technology (CSIR), Hyderabad, India. Tel: 91-40-27193210; Fax: 91-40-27160512*

^*^Author for Correspondence

Dr Utpal Bhadra

Email: [utpal@ccmb.res.in](mailto:utpal@ccmb.res.in); Fax: +91-40-27160591

Dr Manika Pal Bhadra

Email: [manikapb@gmail.com](mailto:manikapb@gmail.com), Fax: +91- 040-2716038

**Materials and Methods**

**Synthesis of DCPTN-PT and characterization**:

**Experimental procedure for the synthesis of triazoles:**

All the six triazoles were synthesized by the reaction between corresponding azide and alkyne in the presence of copper catalyst using the procedure mentioned below.

**Procedure:** To a solution of azide (1 mmol) in ethanol (10 mL) were successively added phenyl acetylene (2 mmol), Cu-turnings (0.2 mmol) and saturated copper sulfate solution (aq.) (0.2 mL) and refluxed for given time (Table 1). After completion of the reaction, the reaction mixture was filtered, concentrated *in vacuo* and purified by silica-gel chromatography to afford the corresponding triazole good yield (Table 1).

**Table 1**: Synthesis of triazoles

*1-(4-(3, 4-Dichlorophenyl)-1, 2, 3, 4-tetrahydronaphthalen-1-yl)-4-phenyl-1H-1, 2, 3-triazole* **(1):**

^1^H NMR (200 MHz, CDCl_3_): *δ* 7.84-7.74 (m, 2H), 7.42 (s, 1H), 7.41-7.16 (m, 7H), 7.03-6.85 (m, 3H), 6.09 (t, *J* = 6.2 Hz, 1H), 4.27 (t, *J* = 6.2 Hz, 1H), 2.56-2.20 (m, 3H), 2.07-1.88 (m, 1H); ^13^C NMR (75 MHz, CDCl_3_): *δ* 147.6, 146.0, 138.6, 133.7, 132.5, 130.6, 130.5, 130.4, 128.8, 128.7, 128.4, 128.0, 127.9, 127.6, 125.6, 118.5, 59.2, 44.2, 29.6, 28.8; MS (ESI) *m/z*: 420 [M+H]^+^; HRMS (ESI): calcd for C_24_H_20_N_3_Cl_2_ ([M+H]^+^): 420.1034. Found 420.1032; IR (KBr) *ν_max_* 3116, 2954, 2881, 1598, 1460, 1384, 1273, 1232, 1028 cm^-1^; [α ]^25^_D_ = +2.1 (*c* 1.0, CHCl_3_); mp 164 ^o^C.

*1-(4-(3, 4-Dichlorophenyl)-1, 2, 3, 4-tetrahydronaphthalen-1-yl)-4-pentyl-1H-1, 2, 3-triazole* **(2):**

^1^H NMR (300 MHz, CDCl_3_): *δ* 7.35 (d, *J* = 8.1 Hz, 1H), 7.24-7.14 (m, 3H), 7.00-6.84 (m, 4H), 6.00 (t, *J* = 6.7 Hz, 1H), 4.23 (t, *J* = 6.7 Hz, 1H), 2.65 (t, *J* = 7.3 Hz, 2H), 2.46-2.34 ( m, 1H), 2.32-2.16 (m, 2H), 2.00-1.86 (m, 1H), 1.72-1.56 (m, 2H), 1.40-1.26 (m, 4H), 0.89 (t, *J* = 6.6 Hz, 3H); ^13^C NMR (75 MHz, CDCl_3_): *δ* 148.3, 146.0, 138.4, 134.0, 132.4, 130.4, 130.1, 128.5, 128.2, 127.9, 127.3, 119.4, 60.2, 58.8, 44.2, 31.3, 29.7, 28.8, 25.6, 22.2, 13.8; MS (ESI) *m/z*: 414 [M+H]^+^; HRMS (ESI): calcd for C_23_H_26_N_3_Cl_2_ ([M+H]^+^): 414.1503. Found 414.1518; IR (neat) *ν_max_*  3133, 3062, 3024, 2930, 2861, 1587, 1515, 1466, 1396, 1313, 1217, 1129, 1045 cm^-1^; [α ]^25^_D_ = -1.89 (*c* 2.0, CHCl_3_).

*Dimethyl 1-(4-(3, 4-dichlorophenyl)-1, 2, 3, 4-tetrahydronaphthalen-1-yl)-1H-1, 2, 3-triazole-4, 5-dicarboxylate* **(3):**

^1^H NMR (200 MHz, CDCl_3_): *δ* 7.37 (d, *J* = 8.0 Hz, 1H), 7.22 (d, *J* = 2.1 Hz, 1H), 7.16-7.07 (m, 2H), 6.95 (dd, *J* = 8.0, 2.1 Hz, 1H), 6.86-6.79 (m, 1H), 6.78-6.70 (m, 1H), 6.27 (t, *J* = 6.5 Hz, 1H), 4.29-4.18 (m, 1H), 3.96 (s, 3H), 3.78 (s, 3H), 2.60-2.36 (m, 3H), 2.06-1.83 (m, 1H); ^13^C NMR (75 MHz, CDCl_3_): *δ* 160.3, 159.4, 145.9, 139.3, 139.0, 132.6, 132.4, 131.0, 130.4, 129.9, 128.5, 128.0, 127.5, 127.1, 60.1, 53.3, 52.4, 44.4, 30.3, 29.2; MS (ESI) *m/z*: 482 [M+Na]^+^; HRMS (ESI): calcd for C_22_H_19_N_3_O_4_Cl_2_Na ([M+Na]^+^): 482.0650. Found 482.0639; IR (KBr) *ν_max_* 3022, 2952, 1735, 1631, 1558, 1456, 1384, 1354, 1281, 1222, 1136, 1060 cm^-1^; [α ]^25^_D_ = +2.3 (*c* 1.0, CHCl_3_); mp 149 ^o^C.

*1-(2-(4-Isobutylphenyl)propyl)-4-phenyl-1H-1, 2, 3-triazole* **(4):**

^1^H NMR (300 MHz, CDCl_3_): *δ* 7.66 (d, *J* = 7.5 Hz, 2H), 7.37-7.21 (m, 3H), 7.17 (s, 1H), 7.08- 7.00 ( m, 4H), 4.56 (dd, *J* = 12.8, 6.7 Hz, 1H), 4.38 (dd, *J* = 12.8, 7.5 Hz, 1H), 3.41-3.27 (m, 1H), 2.44 (d, *J* = 6.7 Hz, 2H), 1.92-1.76 (m, 1H), 1.36 (d, *J* = 6.7 Hz, 3H), 0.89 (d, *J* = 6.7 Hz, 6H); ^13^C NMR (75 MHz, CDCl_3_): *δ* 147.0, 140.5, 139.4, 130.6, 129.4, 128.6, 127.8, 126.7, 125.5, 119.9, 57.2, 44.8, 40.4, 30.0, 22.2, 18.4; MS (ESI) *m/z*: 320 [M+H]^+^; HRMS (ESI) calcd for C_21_H_26_N_3_ ([M+H]^+^): 320.2126. Found 320.2138; IR (KBr) *ν_max_* 3082, 2953, 2866, 2836, 1629, 1586, 1384, 1364, 1226, 1162, 1078, 1020 cm^-1^; [α ]^25^_D_ = +1.1 (*c* 1.0, CHCl_3_); mp 120 ^o^C.

*1-(2-(4-Isobutylphenyl)propyl)-4-pentyl-1H-1, 2, 3-triazole* **(5):**

^1^H NMR (300 MHz, CDCl_3_): *δ* 7.06-6.96 (m, 4H), 6.71 (s, 1H), 4.46 (dd, *J* = 13.5, 7.1 Hz, 1H), 4.29 (dd, *J* = 13.5, 7.5 Hz, 1H), 3.35-3.21 (m, 1H), 2.58 (t, *J* = 7.7Hz, 2H), 2.43 (d, *J* = 7.1 Hz, 2H), 1.91-1.76 (m, 1H), 1.63-1.51 (m, 2H), 1.37-1.21 (m, 7H), 0.94-0.84 (m, 9H); ^13^C NMR (75 MHz, CDCl_3_): *δ* 147.7, 140.4, 139.7, 129.3, 126.6, 120.9, 57.0, 44.8, 40.5, 31.2, 30.0, 29.0, 25.4, 22.3, 22.2, 18.4, 13.8; MS (ESI) *m/z*: 314 [M+H]^+^; HRMS (ESI) calcd for C_20_H_32_N_3_ ([M+H]^+^): 314.2596. Found 314.2592; IR (KBr) *ν_max_*  3114, 3065, 2958, 2928, 2861, 1629, 1589, 1514, 1460, 1383, 1214, 1145, 1054, 1018 cm^-1^; [α ]^25^_D_ = +1.3 (*c* 1.05, CHCl_3_); mp 58 ^o^C.

*Dimethyl 1-(2-(4-isobutylphenyl)propyl)-1H-1, 2, 3-triazole-4, 5-dicarboxylate* **(6):**

^1^H NMR (200 MHz, CDCl_3_): *δ* 7.08-6.92 (m, 4H), 4.80-4.61 (m, 2H), 3.93 (s, 3H), 3.83 (s, 3H), 3.46-3.25 (m, 1H), 2.42 (d, *J* = 7.3 Hz, 2H); 1.93-1.71 (m, 1H), 1.31 (d, *J* = 7.3 Hz, 3H), 0.89 (d, *J* = 6.5 Hz, 6H); ^13^C NMR (75 MHz, CDCl_3_); *δ* 160.2, 158.8, 140.5, 139.4, 138.5, 130.0, 129.4, 126.7, 56.5, 52.9, 52.3, 45.0, 40.5, 30.1, 22.39, 22.31, 18.1; MS (ESI) *m/z*: 360 [M+H]^+^; HRMS (ESI) calcd for C_19_H_26_N_3_O_4_ ([M+H]^+^): 360.1923. Found 360.1918; IR (KBr) *ν_max_* 3133, 2956, 2871, 2838, 1735, 1562, 1469, 1384, 1356, 1320, 1268, 1228, 1181, 1150, 1111, 1059 cm^-1^; [α ]^25^_D_ = +1.1 (*c* 1.05, CHCl_3_); mp 55 ^o^C.

**Biology Experimental procedures:**

**Live animal feeding.**

**Feeding of *Drosophila* larvae:** For equal feeding during preparation of *Drosophila* culture food media in each vial, 3ml of food media was administered with 12 µM of DCPTN-PT compound to make each vial. An equal number of flies 20 flies (10 males + 10 females) were added in each vial for 3 days. Flies were transferred to new vials after rearing them in equal amount of time period. Vials were reared in culture condition for 10 days before third Instar larvae were scarified.

**Human cell Culture:** Human cancer cell lines which has positive Caspase 3, HeLa cells were grown in Dulbecco’s Modified Eagle’s Medium (Sigma Chemical, USA) supplemented with 10% fetal bovine serum and common antibiotics (penicillin, kanamycin, and streptomycin) at 1X concentration. Cells were cultured in a standard humidified atmosphere of 5% CO_2_ at 37°C.

**MTT Assay:** The cell proliferation was carried out by colorimetric assay using 3-(4,5dimethylthiazol-2yl)-2,5 diphenyl-tetrazolium bromide (MTT). The assay was based on reductive capacity to metabolize the tetrazolium salt to blue coloured formazane. The cultured cells were seeded on 96 well micro well plates nearly 15000 cells/well. They were incubated at an optimal concentration of compounds (4µM) in fresh media for 48 hrs. MTT assay was performed after 24 hr time period. Briefly, cells were incubated with 0.5 mg/ml of MTT (Sigma) for 4 hr in a CO2 incubator at 37˚C. After the discard the solution, the purple precipitates were dissolved in DMSO (0.01%) for 20 min at room temperature and the resultant solution was transferred to new 96-well plates. The absorbance was measured at 570 nm using a multimode Reader (Thermo Fisher). Each experiment was performed in triplicate, and the average values were presented by a bar diagram.

**Cell Cycle Analysis:** For FACS assay, 5 X 10^5^ HeLa and Drosophila S2 cells were seeded in 60 mm dish and were allowed to grow for 24h. Compounds (1µM. 2 µM, 3 µM and 4µM) were incubated to the culture media at this point and further incubated for an additional 24h. The cells was harvested with Trypsin-EDTA, fixed with ice-cold 70% ethanol at 4^o^C for 30 min, washed with PBS and later incubated with 1mg/ml RNase A solution (Sigma) at 37^o^C for 30 min. Cells were collected by centrifugation at 2000 rpm for 5 min and further stained with 250 μl of DNA staining solution [10 mg of Propidium Iodide (PI), 0.1mg of trisodium citrate, and 0.03 ml of Triton X-100 were dissolved in 100 ml of sterile Milli Q water at room temperature for 30 min in the dark]. The DNA contents of 20,000 events were measured by flow-cytometer as described the manufacturer protocol. (DAKO CYTOMATION, Beckman Coulter, Brea, CA). Histograms were analyzed using Summit Software.

**RT-PCR Assay**

RNA isolation and cleanup process was carried out using conventional method. To synthesize first-strand cDNA from total RNA, a 20 μl reaction mixture was prepared for each sample. 2 µg of total RNA was diluted in nuclease free water to obtain 20 µl of reaction for each sample mix. This 20 μl of RNA-H2O mix was added to each tube of “RNA to cDNA EcoDry Premix [Clontech, RNA to cDNA EcoDry™ Premix (PT5153-2)]”. All reactions were carried out in eppendorf vapo protect thermal cycler after vigorous mixing of tubes. A condition used during the process was as follows- 42°C for 60 minutes followed by 70°C for 10 minutes to stop the reaction Later PCR reactions were also carried out in eppendorf vapo protect thermal cycler. 1µl of cDNA was mixed with 0.2µM of gene specific forward and reverse primers. Emerald Amp GT PCR Master Mix containing DNA polymerase, optimized reaction buffer, dNTPs, and a density reagent was mixed with each reaction mixture and then reaction volume was adjusted to 10 µl to reach 1X final concentration. All reactions were carried out in triplicates. Standard conditions used during amplification was as follows- 98°C for 1 min, (98°C - 10 sec , 58°C - 30 sec, 72°C – 45 sec)X 30 Cycles, followed by  72°C for 6 min and then holding at 4°C . After completion of reactions, all samples were run at 90V in 1% agarose gel. Finally ethidium bromide stained gel images were captured using Bio-Rad Gel Doc™ XR System. The following cDNA primers of each gene were used for RT PCR amplification.

| drosha-FP | TACGTCTACCCGGAGACACC |
| --- | --- |
| drosha-RP | ATGGTGGTAGCCTTGACTGG |

| pasha-FP | CAATGTGGCACTTCCAATGA |
| --- | --- |
| pasha-RP | CTTCGTCGAGAACCTGGAAC |

Dicer-1 –FP GTGGAACTGCACAGGATCGG

Dicer-1 –RP GTTCCCGGACGTGGTTCATC

and

18S rRNA FP-  5’CCTTATGGGACGTGTGCTTT

18S rRNA RP- 5’CCTGCTGCCTTCCTTAGATG

**TUNEL assay:-**Apoptotic cells were detected by an in situ Apoptosis Detection Kit (TaKaRa), based on terminal deoxynucleotidyl transferase-mediated dUTP nick-end labelling method (TUNEL). TUNEL assay uses terminal deoxynucleotidyl transferase to label 3’-OH ends of DNA fragments, generated during apoptosis process. Apoptotic cells specifically labeled with fluorescein-dUTP with high sensitivity.
S2 cells were grown on poly-L-lysine coated cover slip. Treatments at 2µM, 4µM and 8µM were given after getting 60% confluence. After a course of 24 hour treatment all cells including control were washed with PBS. Then they were fixed using 4% PBS-paraformaldehyde solution at room temperature for 20 minutes. After washing with PBS, 100μl of Permeabilisation Buffer was applied at 4⁰C for 5 minute.  50μl of labeling reaction mixture (a freshly prepared mixture of TdT Enzyme 5 μl and Labeling Safe Buffer 45 μl) was given on each reaction and was incubated in a 37°C humidified chamber for 1.5 hour.  Finally cells were checked under confocal microscope after mounting them in vectashield mounting medium.

**Results**


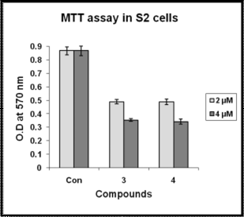


**Figure S1.** **Cell viability (MTT assay) with S2 cell line.**

The compounds (**1 - 6**) were treated at 4 µM concentrations for 24h. MTT assay was conducted. **Compound-2** was found to be effective. Cell viability (MTT assay) with S2 cell line. The compound (DCPTN-PT) was treated at 2µM and 4 µM concentrations for 24h. MTT assay was conducted.


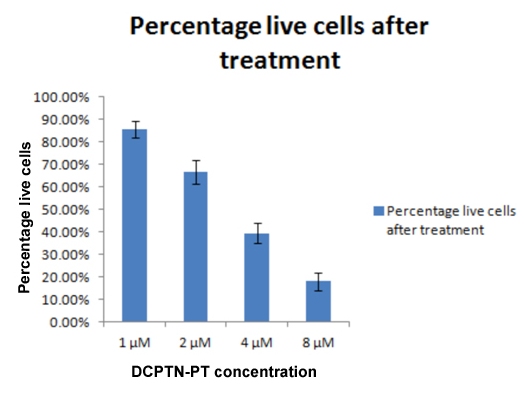


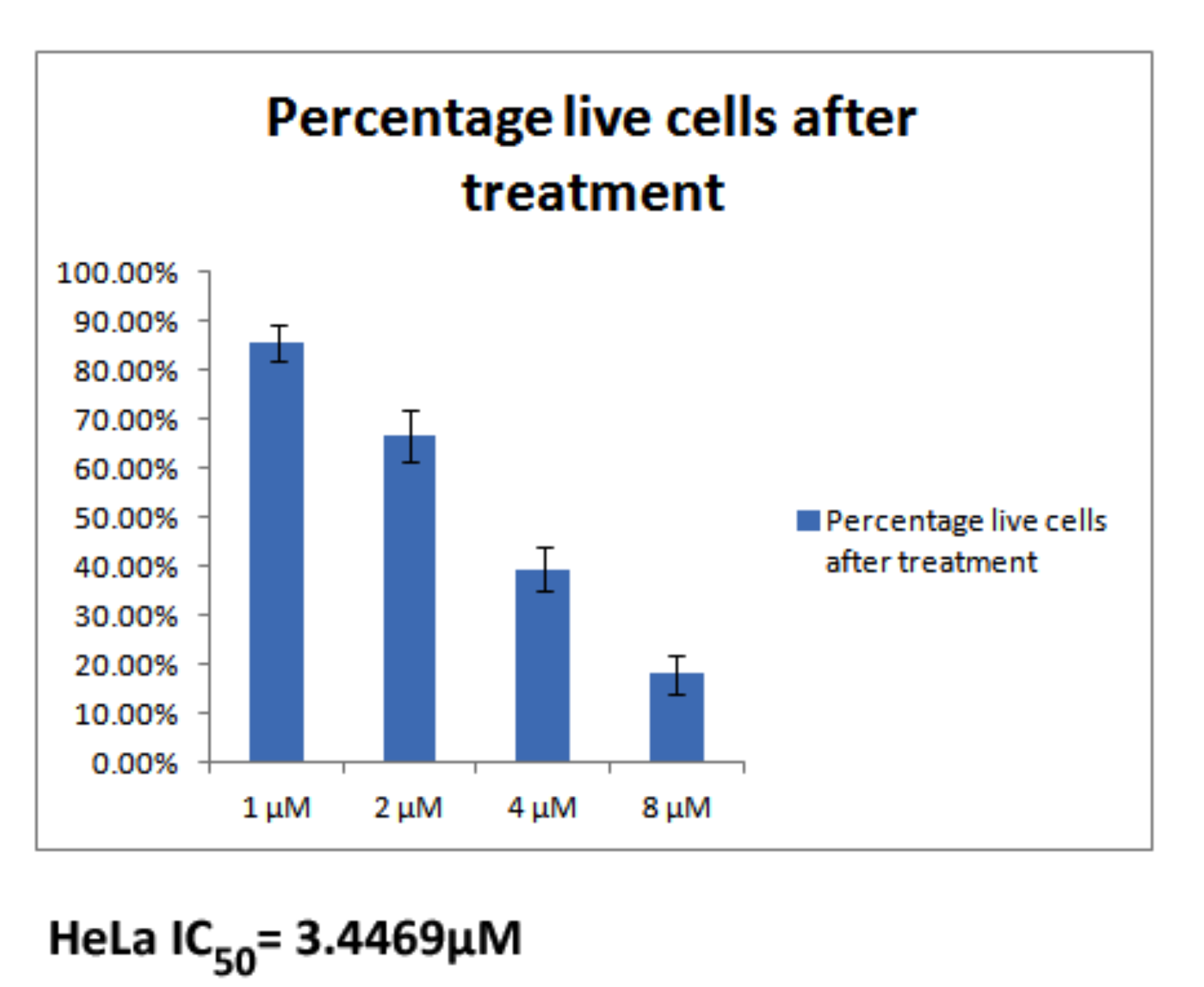


**Figure S2**: IC_50_ value of the HeLa cells was shown by the administered of the DCPTN-PT molecules in different concentrations. IC_50_ represents the concentration of DCPTN-PT that is required for 50% inhibition on cells *in vitro*. The IC_50_ value of the HeLa cells was noted at the bottom.


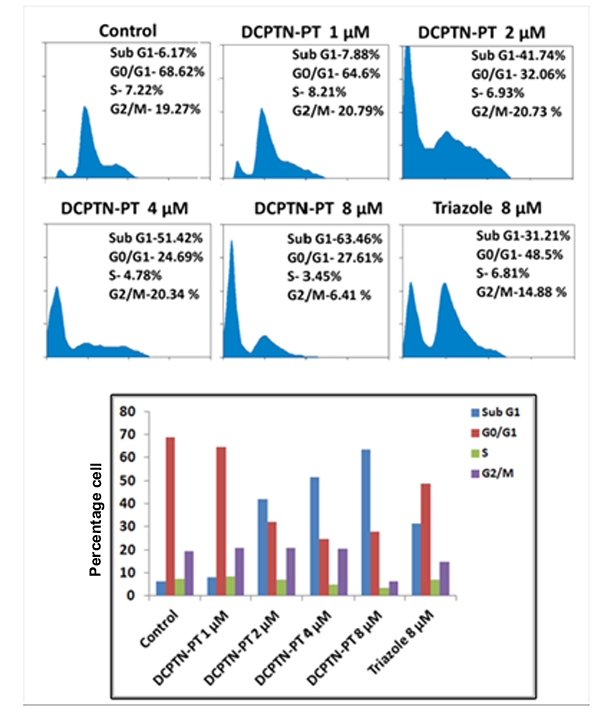


**Figure S3. Flow cytomery assay**

The experiment cell cycle arrest profiles of the HeLa cells were treated with different concentrations of DCPTN-PT compound along with precursor molecules Triazole (8µM) and DMSO (0.01%) containing control media. Cells were accumulated more in G0/G1 phase transition in the cell cycle. The accumulation of Sub G1 cell populations was gradually increased in proportion to the concentrations of DCPTN-PT compound. Bar diagrams at the bottom showed the accumulation of HeLa cells in different stages of cell cycle.

**
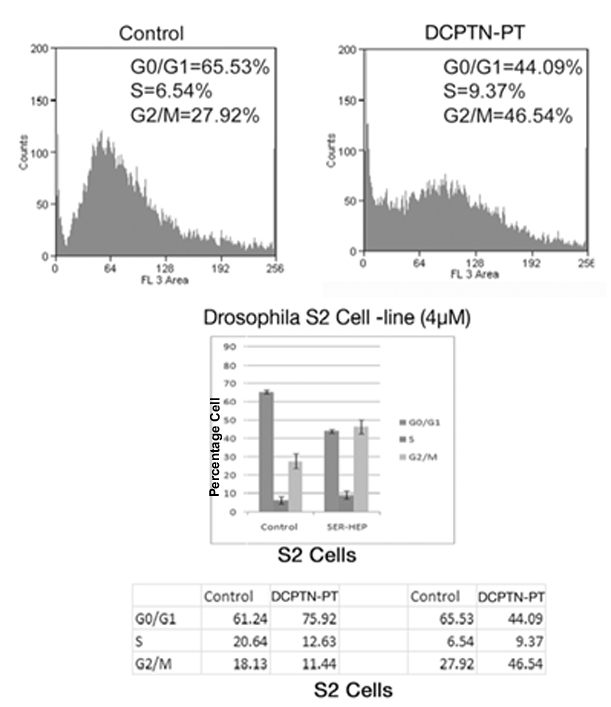
**

**Figure S4** The cell cycle arrest profiles of the Drosophila S2 cells. They were treated optimum concentrations of DCPTN-PT (4µM) compound and DMSO (0.01%) containing control cells. Bar diagrams at the bottom showed the accumulation of Drosophila S2 cells in different phases of the cell cycle. The numerical values of each phage of cell cycle in positive control cells and DCPTN-PT treated cells were also noted.

**
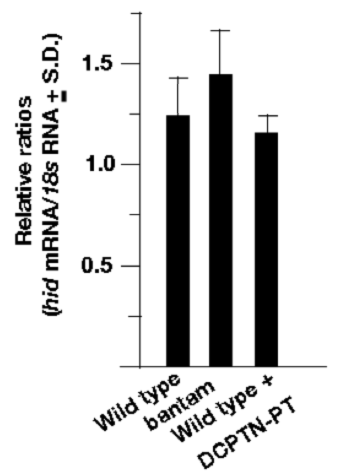
**

**Figure S5.** Quantivative *hid* RNA relative to *18s* RNA was estimated by Real time RT PCR analysis using *hid* and *18s* RNA primers. The relative ratios (Mean ratios *+* S.D) from three separate analyses were plotted as histograms. The genotypes of the larvae were written in the bottom.

**
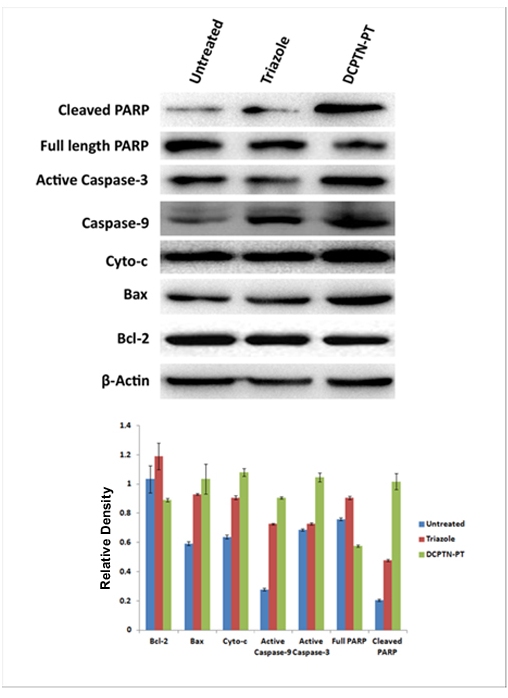
**

**Figure S6**. Quantitative Cell death proteins of the mitochondrial pathways and caspase pathways were shown in HeLa cells. The Hela cells were treated with 4 µM of DCPTN-PT and Triazole (4µM). The untreated cells were used as control. Following the DCPTN-PT exposure, cells were lysed and subjected to Western blot analysis using the indicated primary antibodies. After probing the Blots were reported with ß-Actin antibodies to ensure equal loading and transfer of protein. The results are representative of three separate experiments (Mean ratios + S.E.) were presented in a bar graph. The protein names were noted at the bottom.

**References:**

# 1. Lagos-Quintana, M., Rauhut, R., Lendeckel, W. & Tuschl, T. Identification of novel genes coding for small expressed RNAs. *Science* 294, 853-858.
